# Supplementary material for: Energy Content and Nutrient Profiles of Frequently Consumed Meals in Singapore
Source: Foods. 2021 Jul 19;10(7):1659. doi: 10.3390/foods10071659 (PMC8304763; doi:10.3390/foods10071659)
Supplement: Supplementary file 1 [file foods-10-01659-s001.zip › foods-1282370-SI.pdf]

Supplementary Materials

Table S1: Description of selected commonly consumed Chinese local food

Local Food	Description
Steamed Chicken Rice	Steam chicken rice with chicken breast meat
Roasted Chicken Rice	Chicken, roasted, with skin, served with rice and chilli sauce
Char Kuay Teow	Mixture of flat rice noodles and egg noodles, stir-fried in sweet sauce with eggs, cockles, lap cheong (Chinese sausages), bean sprouts, and Chinese chives.
Yang Zhou Fried Rice	Chinese-style fried rice characterized by its pale rice color adorned with colors coming from bits of meat, seafood like shrimp and crab meat, shiitake mushrooms, peas, carrots, and eggs.
Fried Carrot Cake	Radish cake cubes, stir fried with eggs, preserved radish and then flavoured with seasonings.
Sin Chew Bee Hoon	Rice Vermicelli, stir-fried with light soy sauce, vegetables and a modest sampling of seafood.
Minced Meat Mee Pok	Egg noodles with black mushroom and minced pork, served with chilli sauce
Economical Mee Goreng	Yellow noodles stir fried in sweet sauce with bean sprouts
Lor Mai Kai	Glutinous rice, filled with beans and minced meat
Laska	Thick rice vermicelli served with thick coconut milk gravy with taupok and fishcake
Ban Mian Dry	Flat handmade noodles are tossed in a typical sweet-savoury brown sauce, and topped with braised mushrooms as well as minced pork.
Steamed Chicken Noodle	Egg noodles with steamed chicken breast meat
Fried Hokkien Mee	Fried mixture of yellow noodle and thick rice vermicelli, with added prawn and cuttlefish
Char Siew Wanton Noodles	Minced pork dumpling, boiled, with noodles, sliced char siew, chye sim and gravy
Dumpling You Mian	Handmade noodles cooked in soup typically, and added with ingredients such as minced pork, anchovies, mushrooms, spinach and dumplings.

Table S2: Description of selected commonly consumed Malay local food

Local Food	Description
Ayam Penyet	Smashed fried chicken thigh/drumstick, beancurd, and cucumber served with white rice and sambal chili.
Nasi Kampung Goreng	Cooked rice with eggs, mixed vegetables and tomato sauce, stir-fried, Malay style
Nasi Lemak	Rice cooked in coconut milk, wrapped inside a banana leaf with fried egg, anchovies, chilli sauce and fried chicken wing.
Ikan Penyet	Fried pomfret/cat fish, beancurd, and cucumber served with white rice and sambal chili.
Nasi Ambang	Fragrant rice dish that consists of - but is not limited to- steamed white rice, chicken curry/ chicken stewed in soy sauce, beef or chicken rendang, sambal goreng (lit. fried sambal; a mildly spicy stir-fried stew commonly made with firm tofu, tempeh, and long beans) urap, bergedel, and serunding..
Goreng Pisang (Banana Fritters)	Snack made of banana or plantain, covered in batter or not, being deep fried in hot cooking oil
Tahu Goreng	Deep fried tofu garnished with bean sprouts, cucumber and scallion. Topped with a thick sauce is prepared with shallots, garlic, chili peppers, shrimp paste, soy sauce and tamarind juice.
Mee Bandung	Yellow noodles with spicy,sweet and sour soup.
Mee Bakso	Indonesian beef meatballs served with noodles and soup
Lontong	Rice cakes in a coconut based soup such as sayur lodeh containing shrimp and vegetables like chopped cabbage, turnip and carrots.
Mee Siam	Thin, white rice noodles, hard boiled egg and dried beancurd, served in tangy gravy
Mee Soto	Yellow noodles with shredded chicken in spicy soup
Kentang Ball with Rice Cube	Deep fried mashed potato about the size of a cricket ball and rice cakes in a spicy chicken soup with bean sprouts
Soto Ayam	Soup containing shredded chicken, bean sprouts and rice cubes
Mee Rebus	Thick, yellow egg noodles served in thick spicy gravy together with hard boiled egg

Table S3: Description of selected commonly consumed Indian local food

Local Food	Description
Original Appam	Type of pancake, originating from the Indian subcontinent, made with fermented rice batter and coconut milk.
Egg Appam	Type of pancake, originating from the Indian subcontinent, made with fermented rice batter and coconut milk, with an addition of egg
Roti Prata	Thick, flat, round dough made with wheat flour, sugar, salt and water, fried with ghee
Egg Prata with Chicken Curry	South Indian flatbread that is crisp and flaky on the outside, but soft on the inside with egg as filling. Paired with chicken curry
Boneless Mutton Briyani	Rice cooked with ghee and spices, served with spicy mutton
Poori Set	Indian fried bread made from whole wheat flour and are served with side potato dish.
Marsala Thosai	Wafer filled with potato, long bean, carrot, pea, green chilli and Indian spice
Naan	Indian leavened bread made of flour, plain yoghurt, egs and ghee/butter, pan fried
Chapatti Set with Potato	Flat round pancake made of whole wheat flour or atta flour, ghee and water, pan-fried served with potato as side dish
Putu Mayam	Thin rice noodles with grated coconut and red sugar
Chapatti Set with Potato Marsala	Flat round pancake made of whole wheat flour or atta flour, ghee and water, pan-fried served with curry potato as side dish
Idli Set	Small, fluffy, spherical bread with tapered edges, made with ground rice and urad dahl and served with curry.
Egg Thosai	Round, pancake-like bread,made with fermented rice and gram batter filled with egg.
Vegetable Briyani	Rice cooked with fried carrot, french bean, pea,corn, onion, tomato, cinnamon, cardimon, star anise
Veg Set Meal (Briyani Rice)	Cooked rice with ghee or oil and yellow food colouring and paired with vegetables.

Table S4: Spike Recovery of Chinese Ethnic Local Food (Fried Carrot Cake)

	Unit	Spiked	Sample	Found	Recovery (%)
Na	mg/L	50	13.34	63.04	99.39
Mg	mg/L	5	0.31	5.24	98.55
K	mg/L	50	2.62	49.88	94.53
Ca	mg/L	50	1.00	48.65	95.30
Mn	mg/L	0.05	0.00	0.05	105.90
Fe	mg/L	5	0.05	5.09	100.68

Table S5: Spike Recovery of Malay Ethnic Local Food (Ayam Penyet)

	Unit	Spiked	Sample	Found	Recovery (%)
Na	mg/L	50	10.33	58.81	96.95
Mg	mg/L	5	0.80	5.77	99.33
K	mg/L	50	5.44	51.89	92.90
Ca	mg/L	50	1.99	49.06	94.13
Mn	mg/L	0.05	0.01	0.07	106.08
Fe	mg/L	5	0.03	5.14	102.16

Table S6: Spike Recovery of Indian Ethnic Local Food (Mutton Briyani)

	Unit	Spiked	Sample	Found	Recovery (%)
Na	mg/L	50	19.70	67.15	94.91
Mg	mg/L	5	0.80	5.68	97.62
K	mg/L	50	7.29	53.50	92.41
Ca	mg/L	50	0.67	48.27	95.20
Mn	mg/L	0.05	0.01	0.06	92.42
Fe	mg/L	5	0.05	5.11	101.28

Figure S1: Mineral contents of the commonly consumed Chinese cuisines (calculated based on portion size). a) Sodium; b) Magnesium; c) Potassium; c) Calcium; d) Manganese; e) Iron.

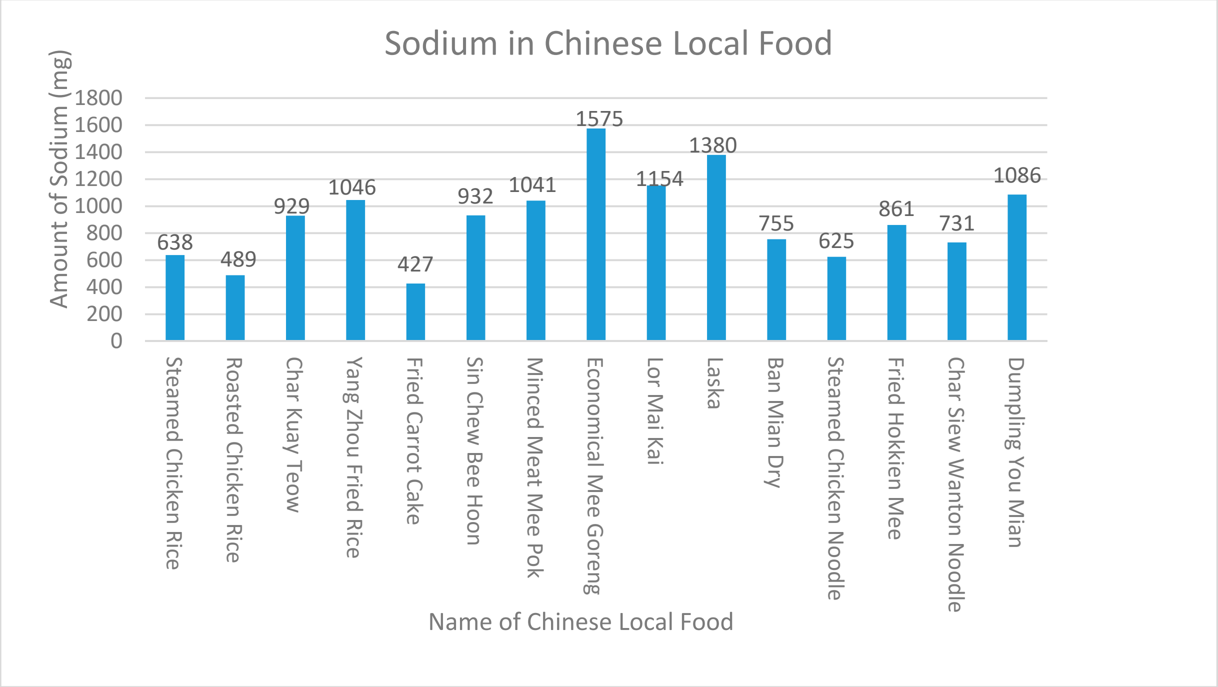

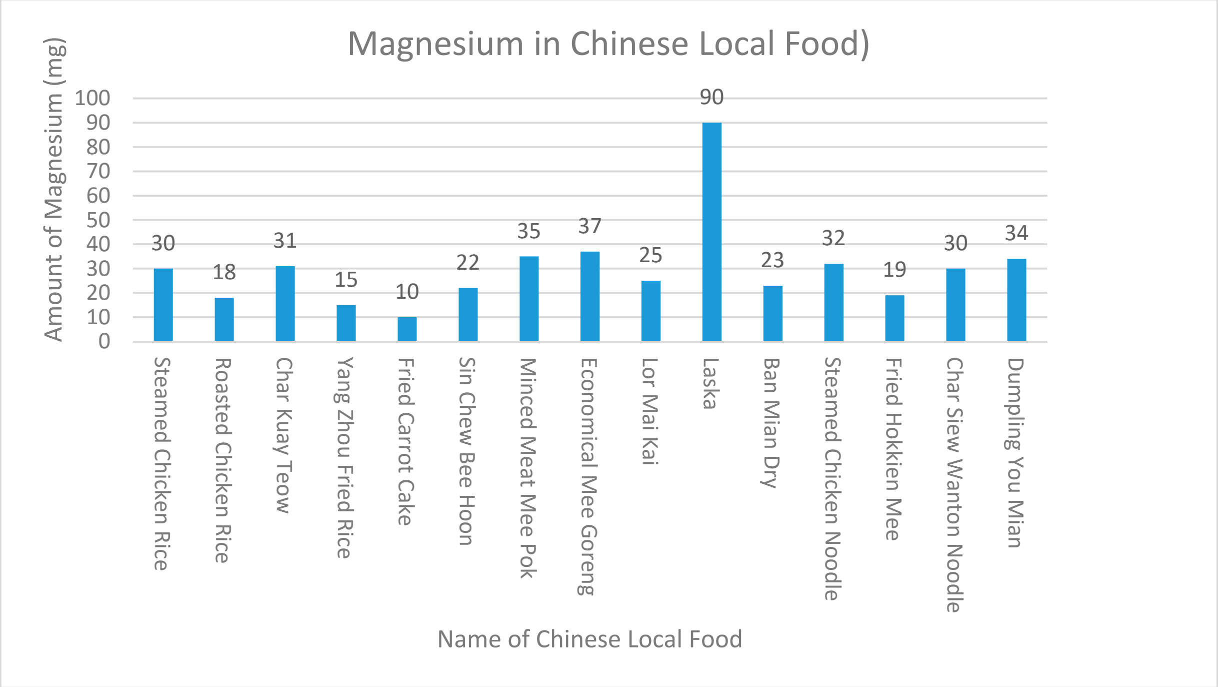

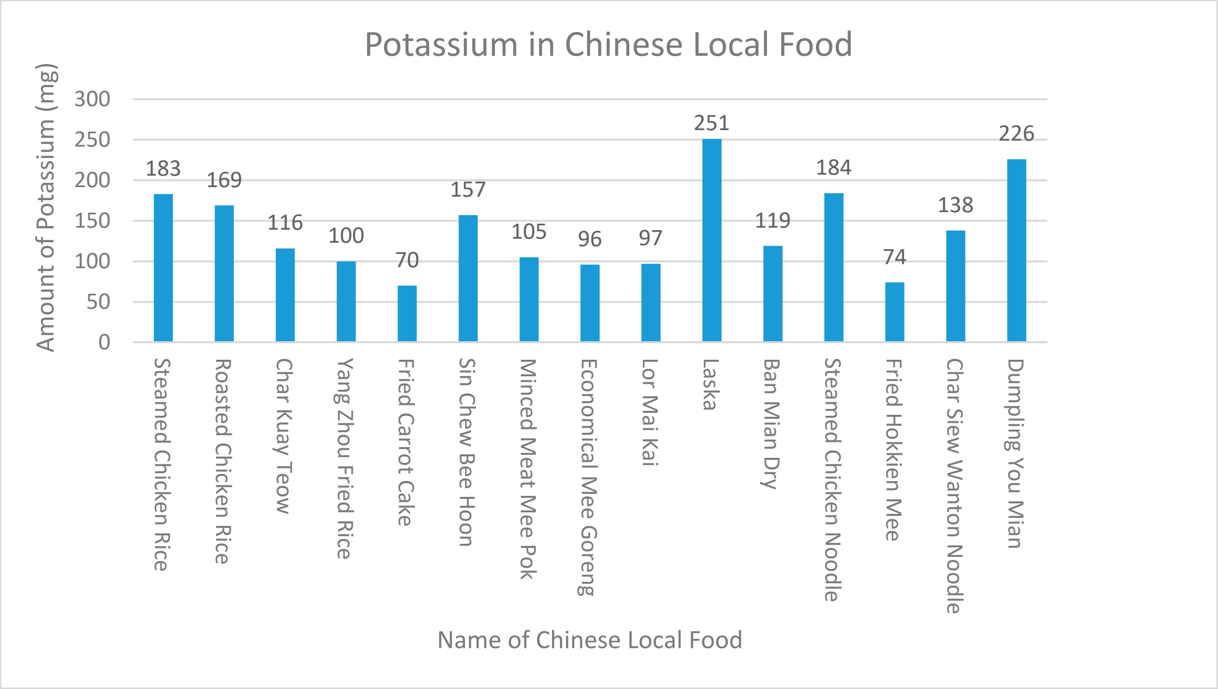

Potassium in Chinese Local Food
Amount of Potassium (mg)
300
250
200
150
100
50
0
183
169
116
100
70
157
105
96
97
251
119
184
74
138
226
Steamed Chicken Rice
Roasted Chicken Rice
Char Kuay Teow
Yang Zhou Fried Rice
Fried Carrot Cake
Sin Chew Bee Hoon
Minced Meat Mee Pok
Economical Mee Goreng
Lor Mai Kai
Laska
Ban Mian Dry
Steamed Chicken Noodle
Fried Hokkien Mee
Char Siew Wanton Noodle
Dumpling You Mian
Name of Chinese Local Food

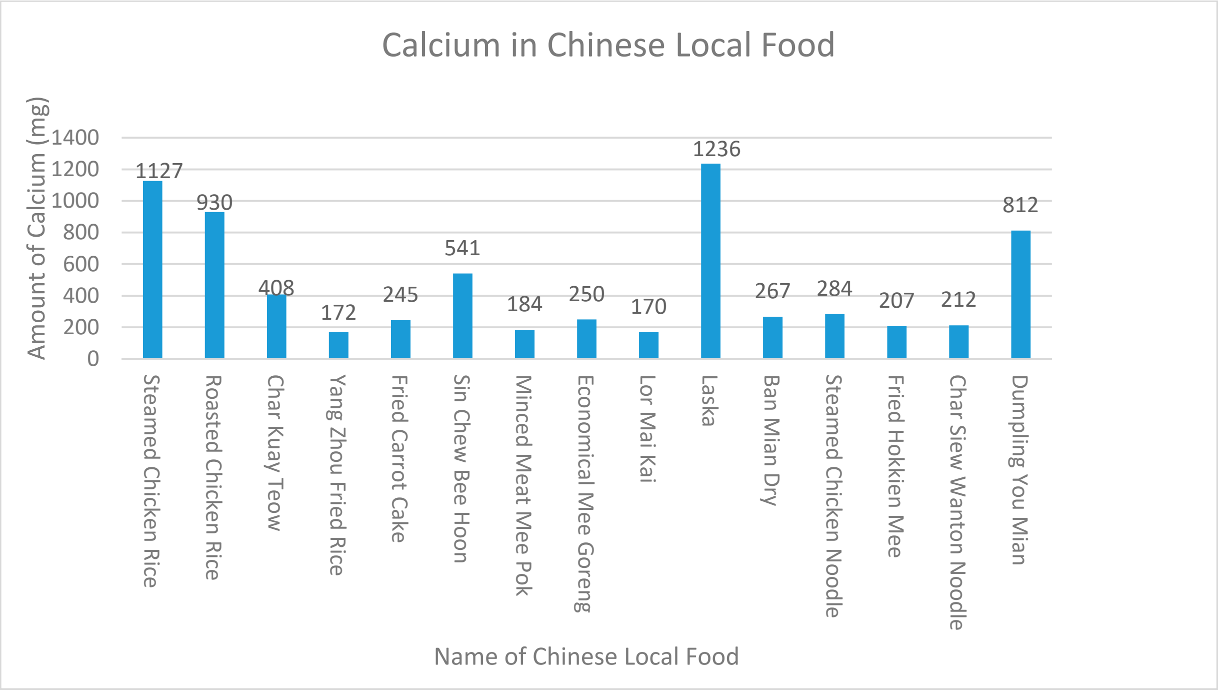

Calcium in Chinese Local Food
Amount of Calcium (mg)
1400
1200
1000
800
600
400
200
0
1127
930
408
172
245
541
184
250
170
1236
267
284
207
212
812
Steamed Chicken Rice
Roasted Chicken Rice
Char Kuay Teow
Yang Zhou Fried Rice
Fried Carrot Cake
Sin Chew Bee Hoon
Minced Meat Mee Pok
Economical Mee Goreng
Lor Mai Kai
Laska
Ban Mian Dry
Steamed Chicken Noodle
Fried Hokkien Mee
Char Siew Wanton Noodle
Dumpling You Mian
Name of Chinese Local Food

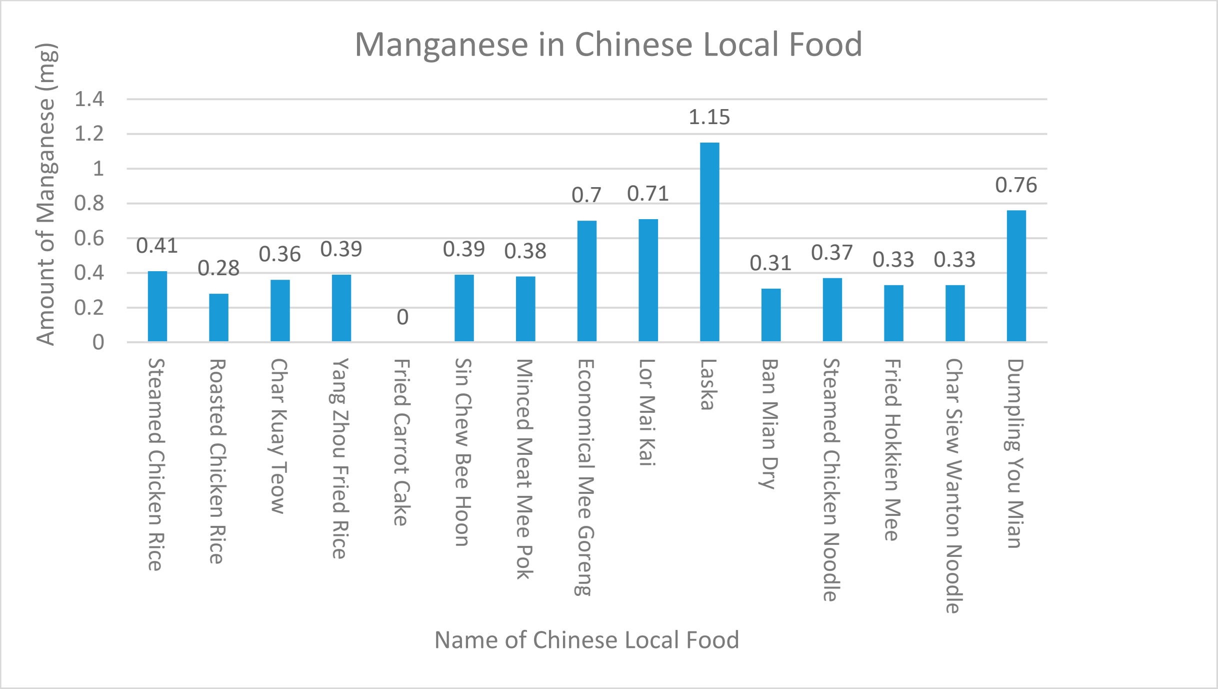

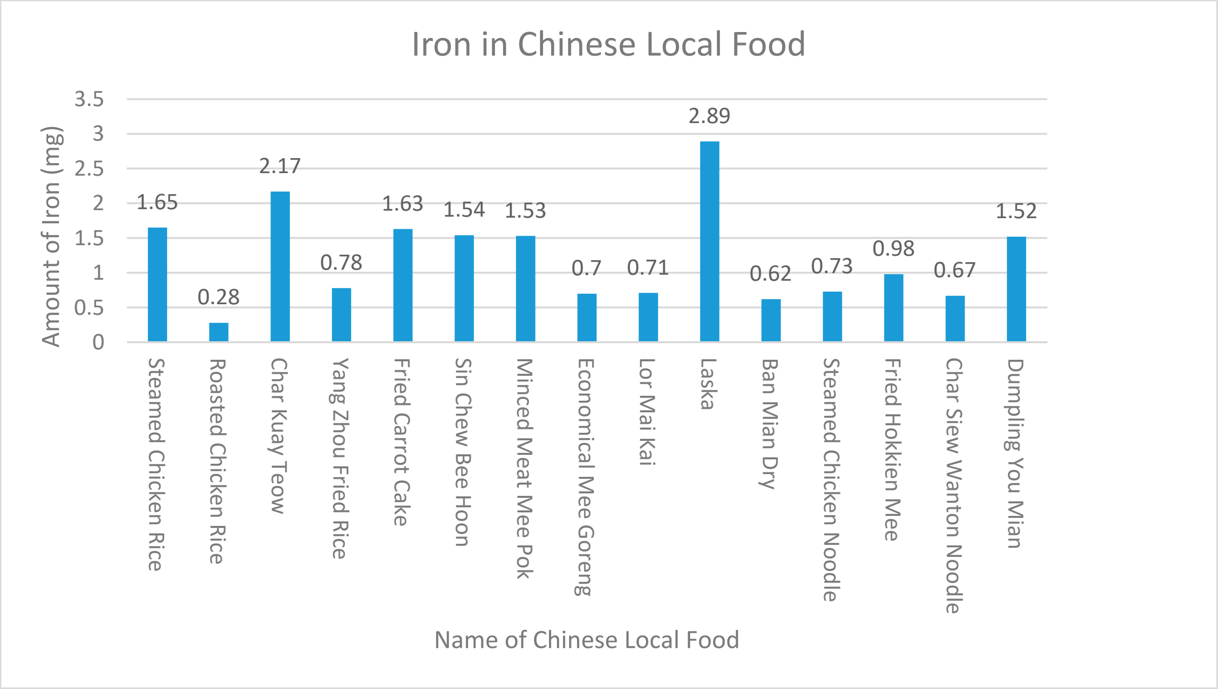

To calculate the mineral content of the food in mg/100g, you can use the value and divide by (portion size/100)

Figure S2: Mineral contents of the commonly consumed Malay cuisines (calculated based on portion size). a) Sodium; b) Magnesium; c) Potassium; c) Calcium; d) Manganese; e) Iron.

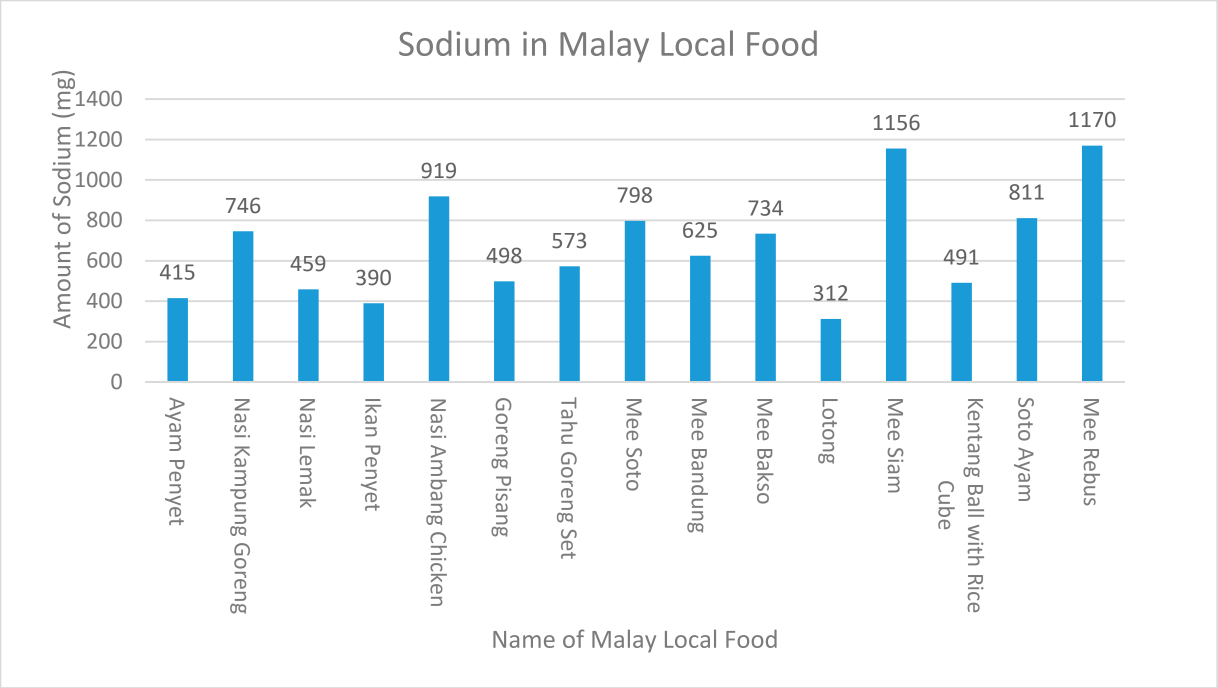

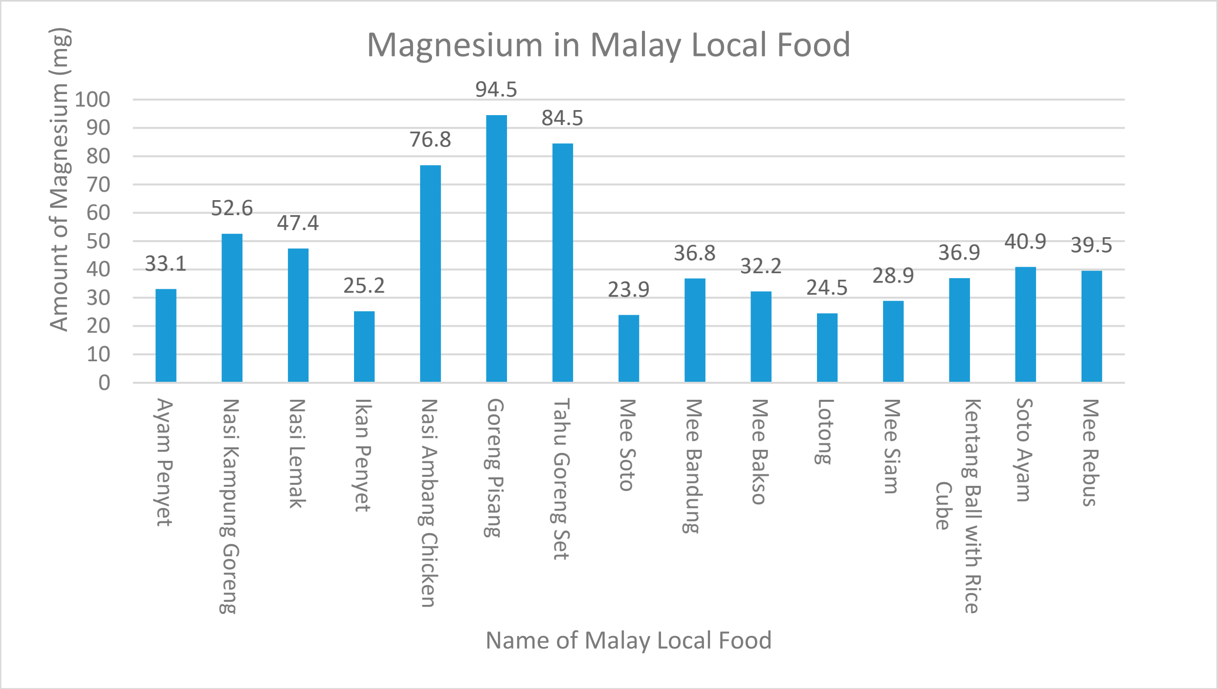

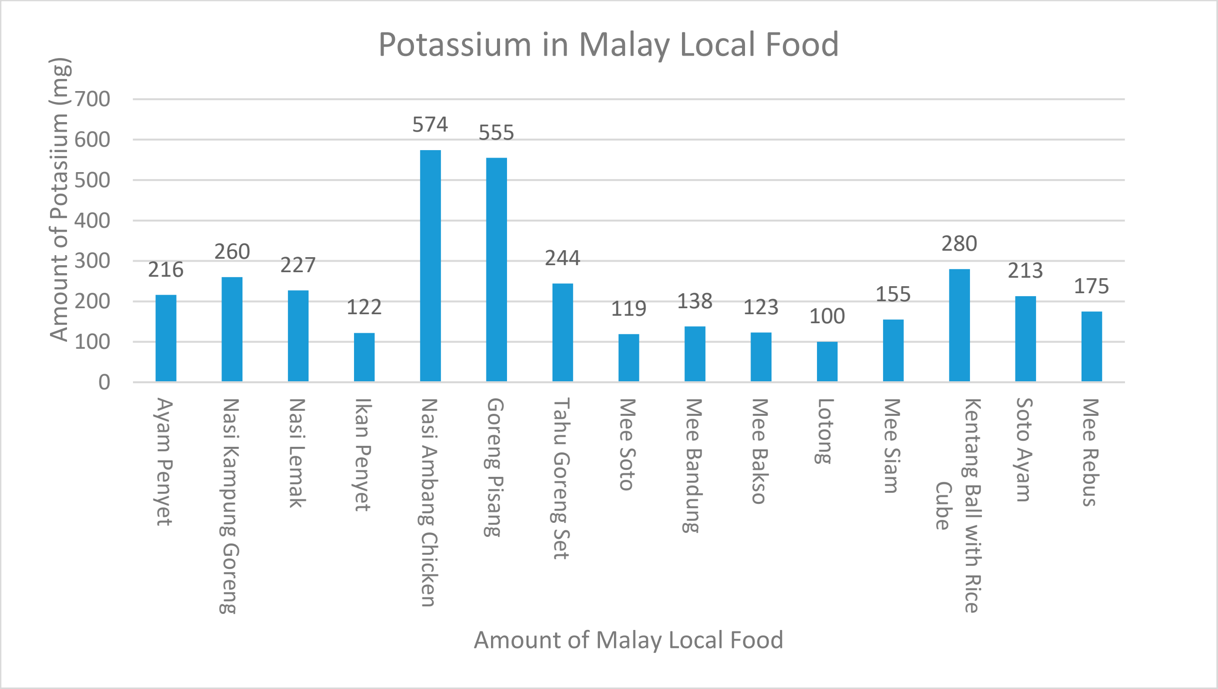

Potassium in Malay Local Food
Amount of Potasiium (mg)
700
600
500
400
300
200
100
0
216
260
227
122
574
555
244
119
138
123
100
155
280
213
175
Ayam Penyet
Nasi Kampung Goreng
Nasi Lemak
Ikan Penyet
Nasi Ambang Chicken
Goreng Pisang
Tahu Goreng Set
Mee Soto
Mee Bandung
Mee Bakso
Lotong
Mee Siam
Kentang Ball with Rice Cube
Soto Ayam
Mee Rebus
Amount of Malay Local Food

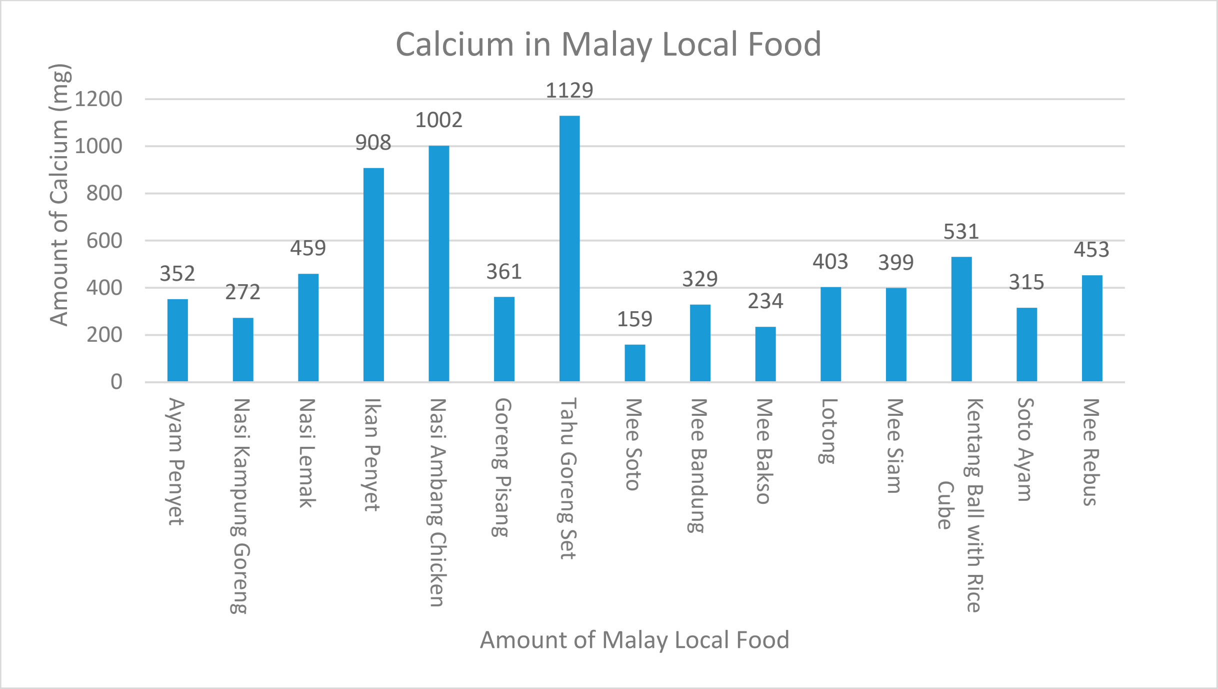

Calcium in Malay Local Food
Amount of Calcium (mg)
1200
1000
800
600
400
200
0
352
272
459
908
1002
361
1129
159
329
234
403
399
531
315
453
Ayam Penyet
Nasi Kampung Goreng
Nasi Lemak
Ikan Penyet
Nasi Ambang Chicken
Goreng Pisang
Tahu Goreng Set
Mee Soto
Mee Bandung
Mee Bakso
Lotong
Mee Siam
Kentang Ball with Rice Cube
Soto Ayam
Mee Rebus
Amount of Malay Local Food

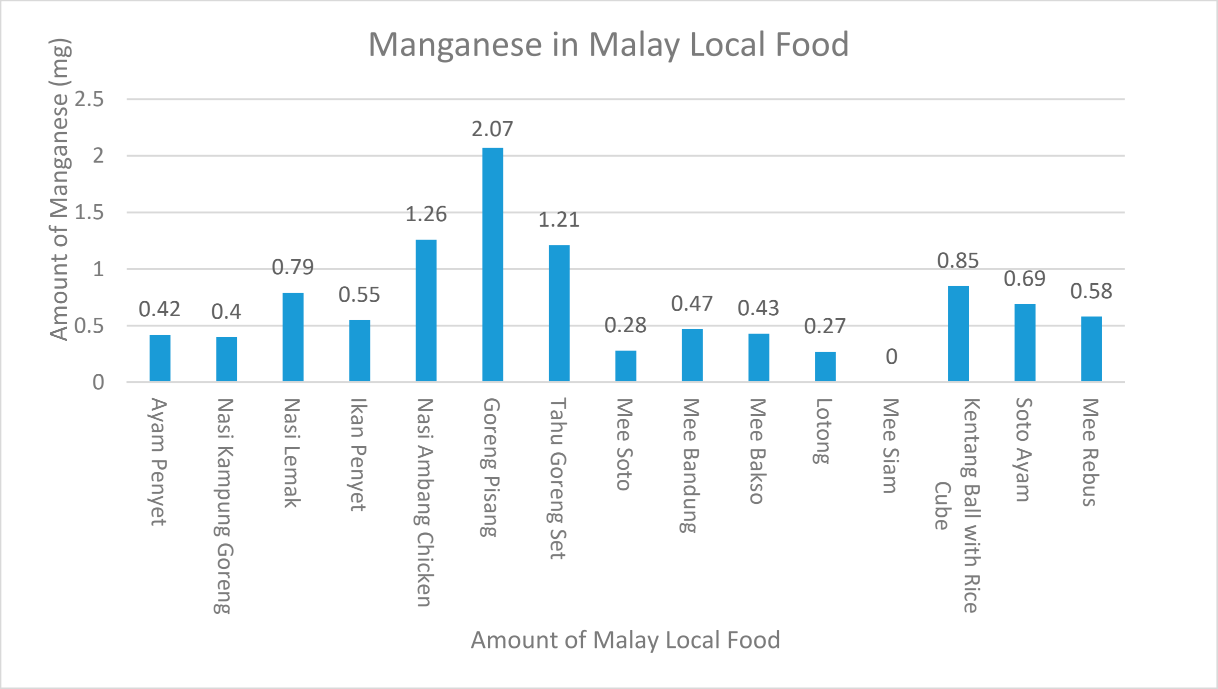

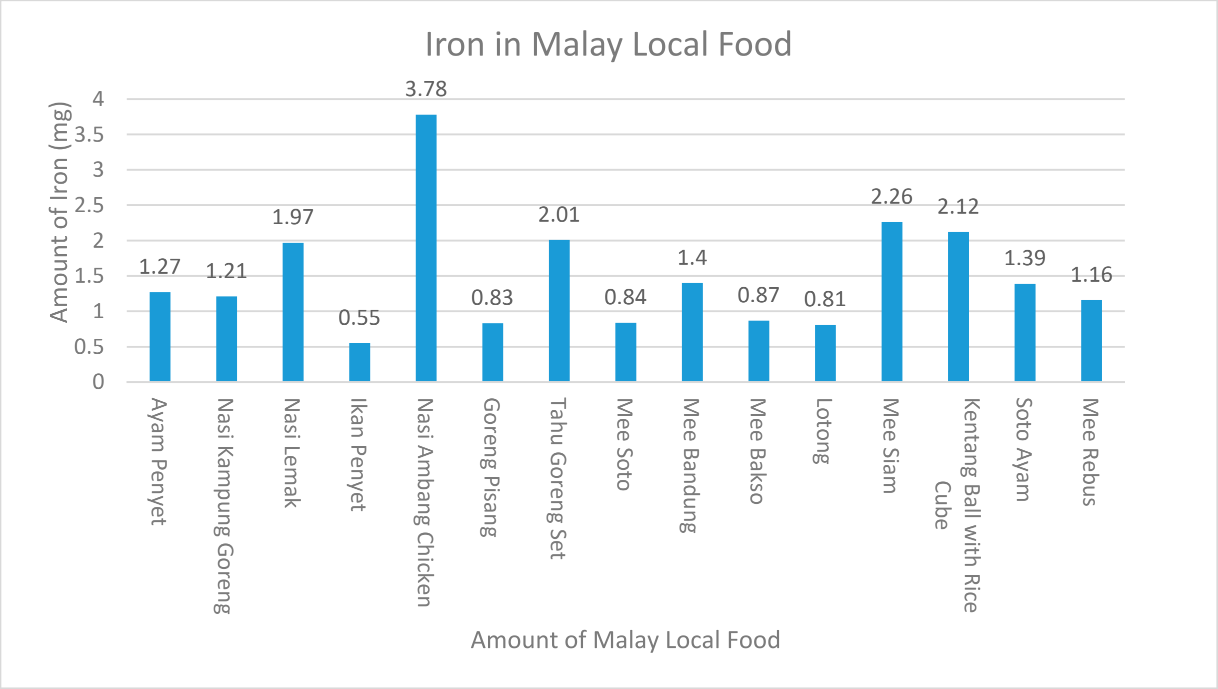

To calculate the mineral content of the food in mg/100g, you can use the value and divide by (portion size/100)

Figure S3: Mineral contents of the commonly consumed Indian cuisines (calculated based on portion size). a) Sodium; b) Magnesium; c) Potassium; c) Calcium; d) Manganese; e) Iron

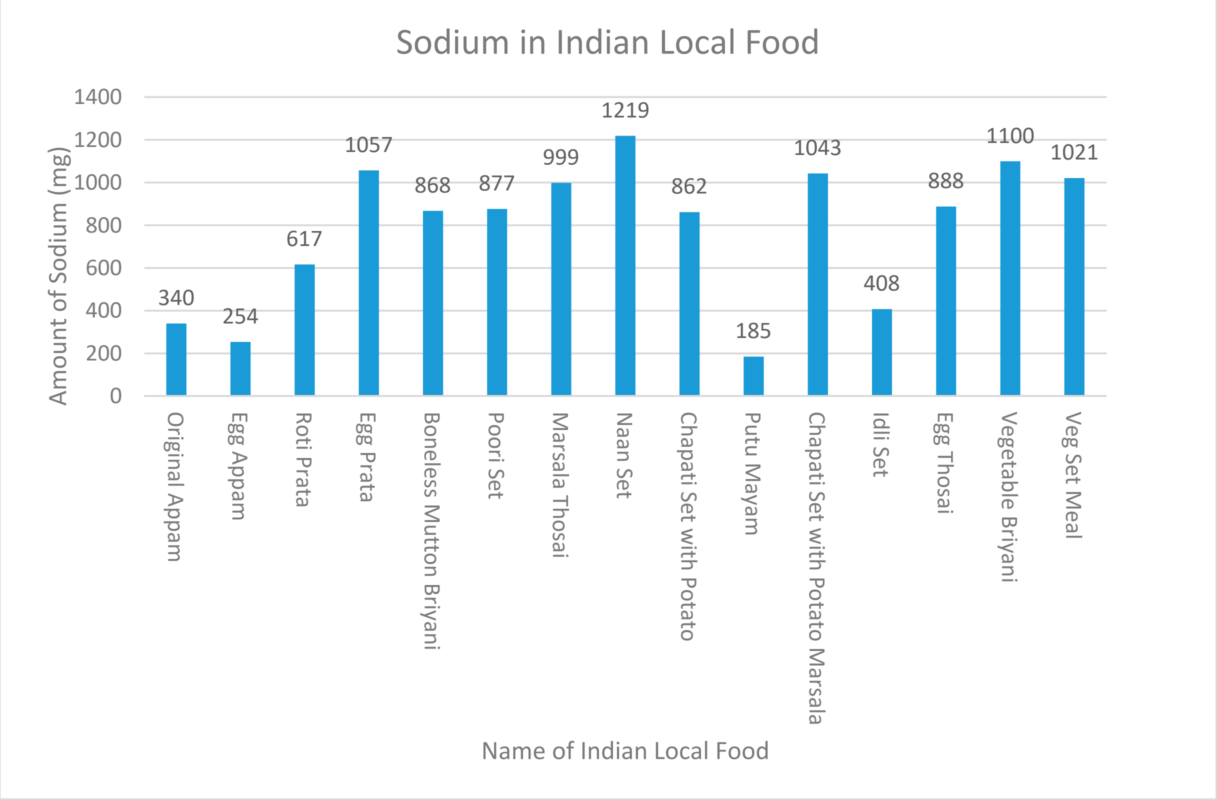

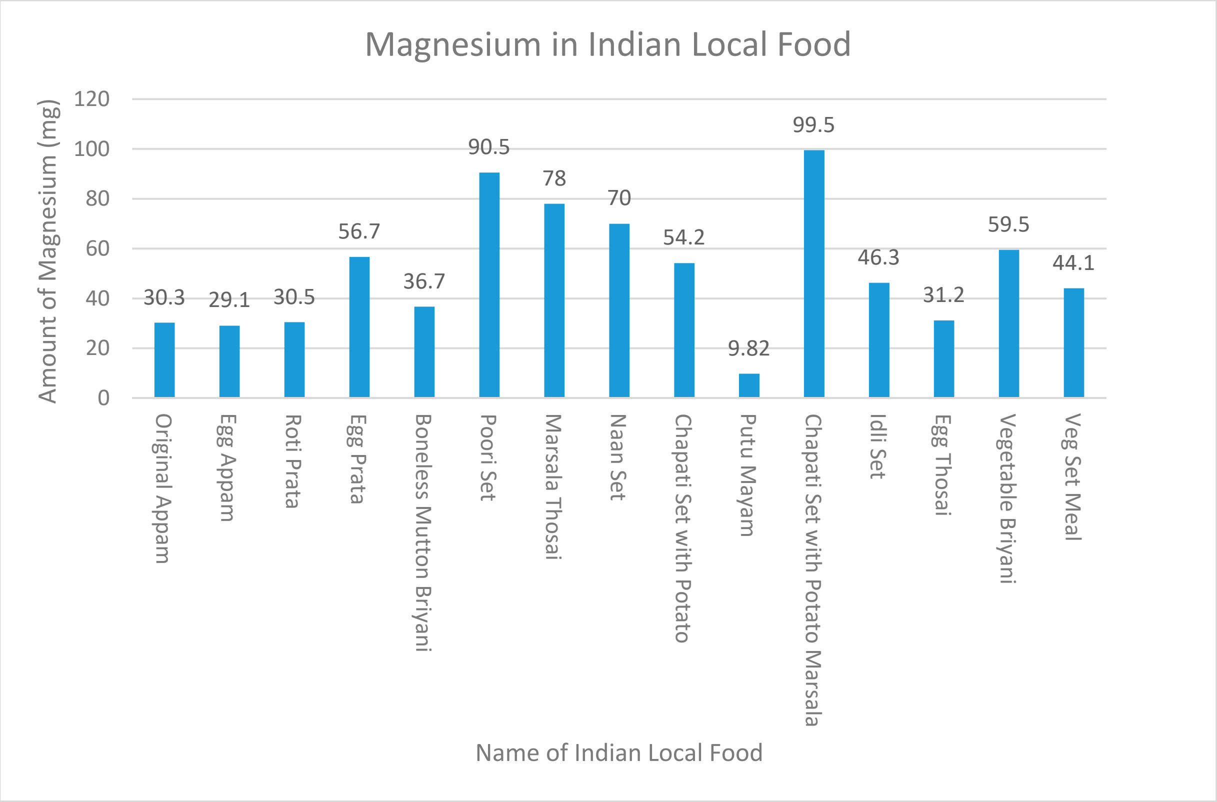

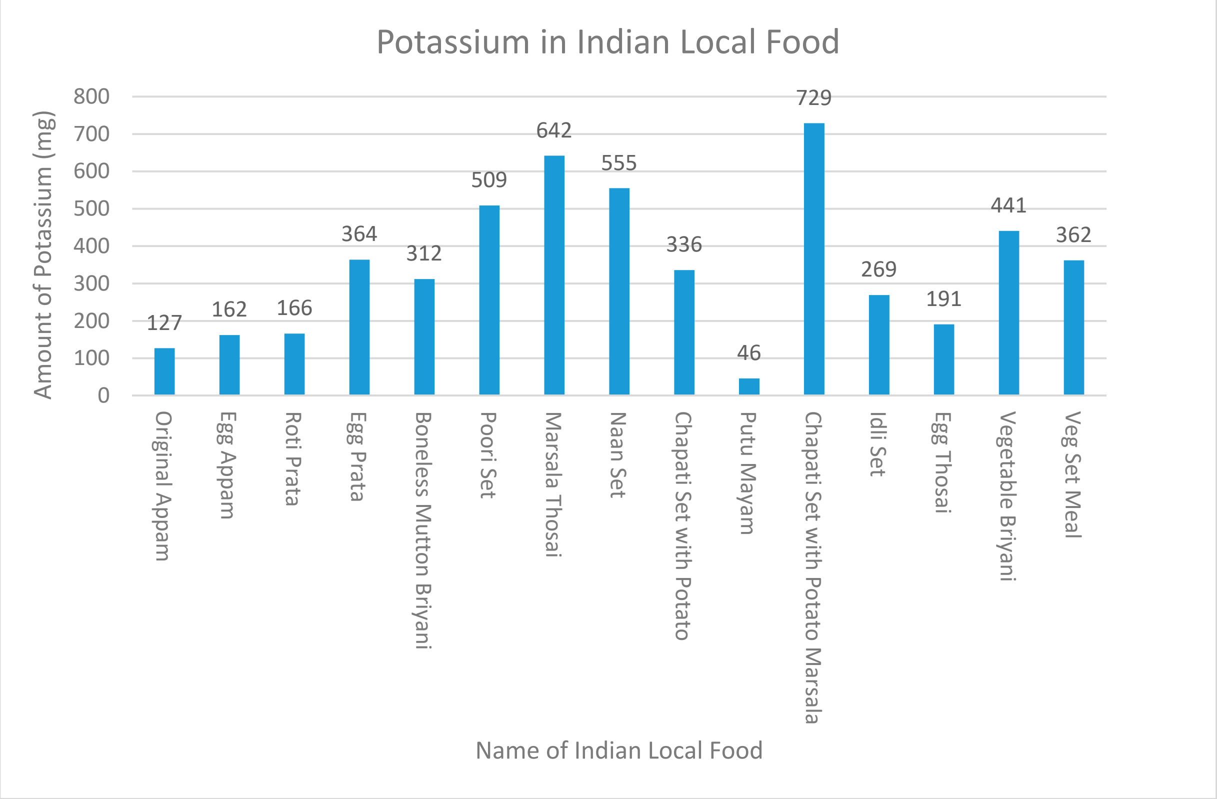

Potassium in Indian Local Food
Amount of Potassium (mg)
800
700
600
500
400
300
200
100
0
127
162
166
364
312
509
642
555
336
46
729
269
191
441
362
Original Appam
Egg Appam
Roti Prata
Egg Prata
Boneless Mutton Briyani
Poori Set
Marsala Thosai
Naan Set
Chapati Set with Potato
Putu Mayam
Chapati Set with Potato Marsala
Idli Set
Egg Thosai
Vegetable Briyani
Veg Set Meal
Name of Indian Local Food

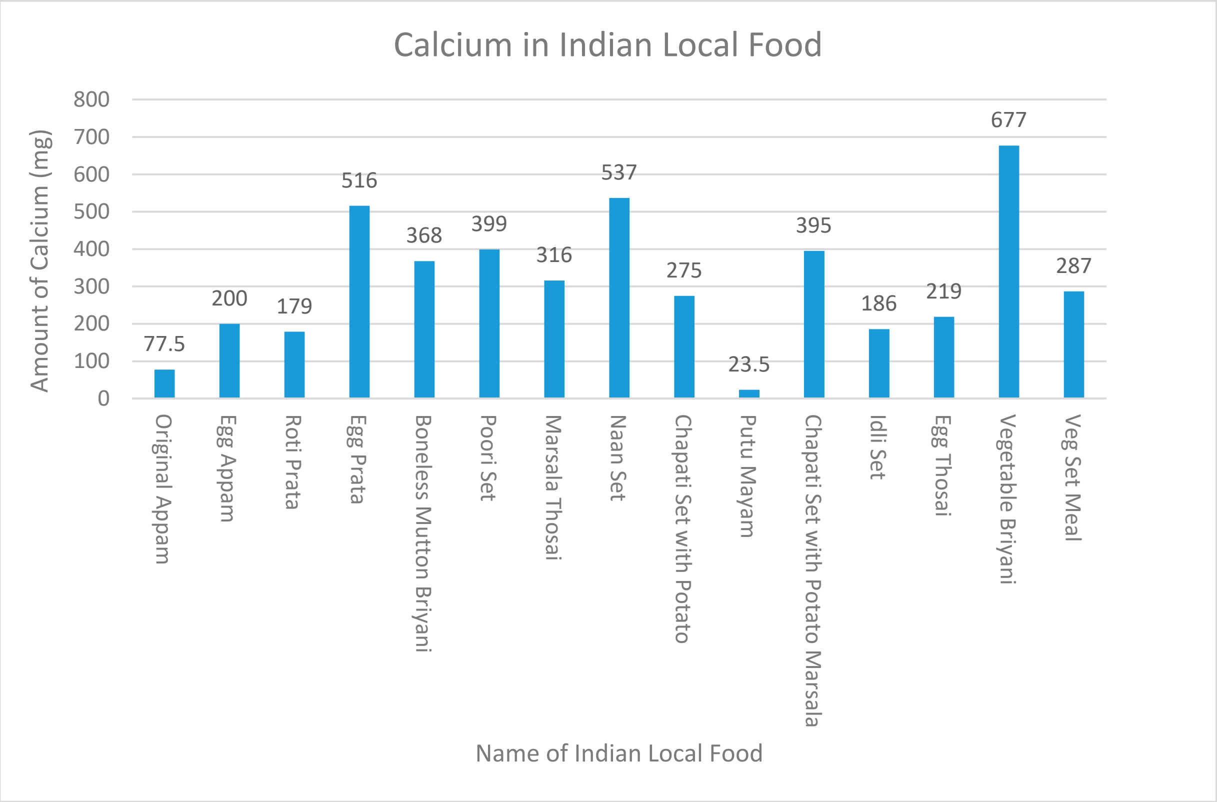

Calcium in Indian Local Food
Amount of Calcium (mg)
800
700
600
500
400
300
200
100
0
77.5
200
179
516
368
399
316
537
275
23.5
395
186
219
677
287
Original Appam
Egg Appam
Roti Prata
Egg Prata
Boneless Mutton Briyani
Poori Set
Marsala Thosai
Naan Set
Chapati Set with Potato
Putu Mayam
Chapati Set with Potato Marsala
Idli Set
Egg Thosai
Vegetable Briyani
Veg Set Meal
Name of Indian Local Food

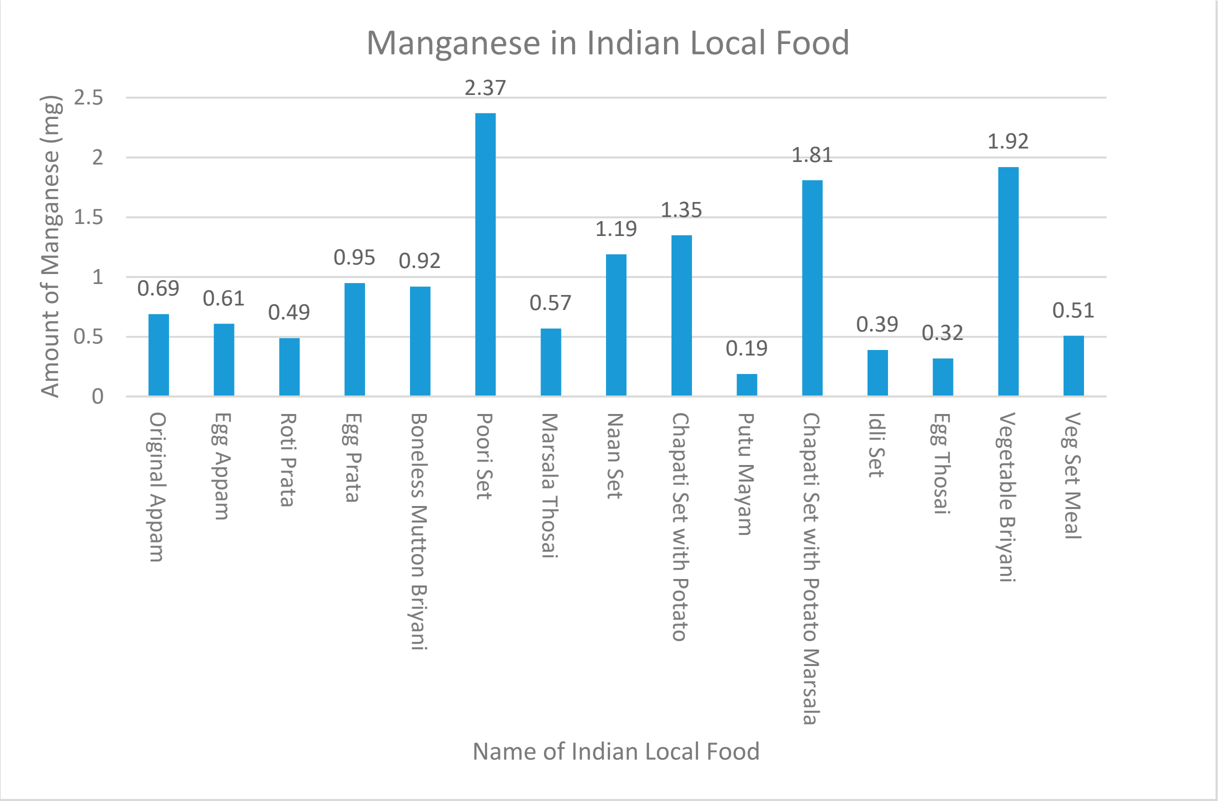

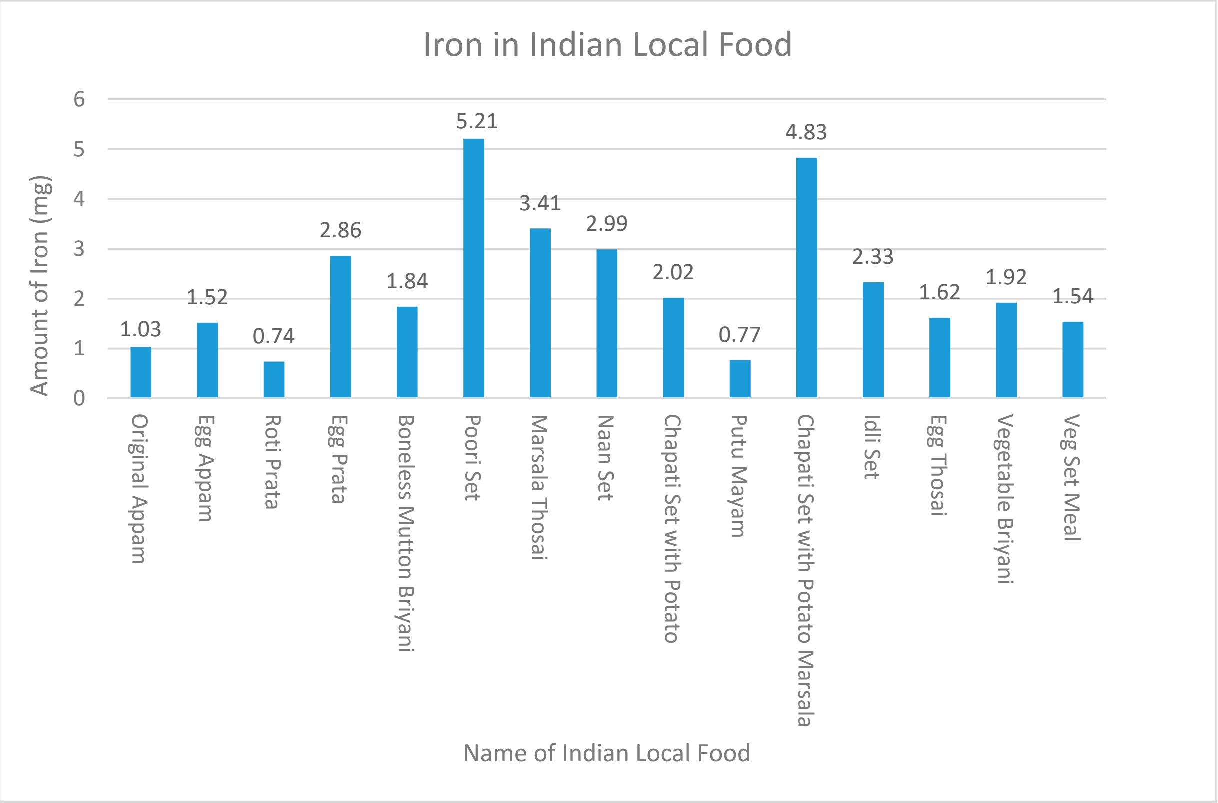

To calculate the mineral content of the food in mg/100g, you can use the value and divide by (portion size/100)

Table S7: Recommended daily allowances of iron, sodium [14]

	Age Group	Iron (mg)	Sodium (mg)
en	18- <30 yrs old	8	2000
	30- <60 yrs	8	2000
	< 60 yrs	8	2000
**Average (Men)**		**8**	**2000**
Women	18- <30 yrs old	18	2000
	30- <60 yrs	18	2000
	< 60 yrs	8	2000
	Pregnant	27	2000
	Lactating	27	2000
**Average (Women)**		**18**	**2000**

Table S8: Recommended daily allowances of calcium for adults [14].

	Age Group	Calcium (mg)
Men	19- 50 yrs old	800
	51 yrs old and above	1000
**Average (Men)**		**1000**
Women	19-50 yrs old	800
	51 yrs old and above	1000
	Pregnant	1000
	Lactating	1000
**Average (Women)**		**1000**

Table S9: Recommended daily allowances of macronutrients for adults [15]

	Age Group	Energy (kcal)	Protein (g)	Total Fat (g)	Carbohydrates (g)
Men	18-29 yrs old	2721		90.7	408.2
	30-39 yrs old	2606		86.9	390.9
	40-49 yrs old	2606	1.07g/kg	86.9	390.9
	50-59 yrs old	2606		86.9	390.9
	60-69 yrs old	2249		75	337.3
**Average (Men)**		**2595**	**76.3**	**86.5**	**389.3**
Women	18-29 yrs old	2081		69.4	312.1
	30-39 yrs old	2048		68.3	307.1
	40-49 yrs old	2048	1.07g/kg	68.3	307.1
	50-59 yrs old	2048		68.3	307.1
	60-69 yrs old	1876		62.5	281.5
**Average (Women)**		**2038**	**62.6**	**67.9**	**305.7**
